# Bovine-Derived *Acinetobacter indicus* Co-Harboring Chromosomal *tet*(X3) and Plasmid-Located *tet*(X4) Isolated from Henan, China

**DOI:** 10.3390/microorganisms14030541

**Published:** 2026-02-26

**Authors:** Qing Wang, Guonian Dai, Yanhua Qiu, Yaxin Zhou, Jing Xu, Weiwei Wang, Jiyu Zhang

**Affiliations:** 1 Lanzhou Institute of Husbandry and Pharmaceutical Sciences, Chinese Academy of Agricultural Sciences, Lanzhou 730050, China; qingwang880826@163.com (Q.W.); dai940910@163.com (G.D.); qyhranglin@163.com (Y.Q.); zhouyaxin@caas.cn (Y.Z.); xujing20122012@163.com (J.X.); 2 College of Veterinary Medicine, Gansu Agricultural University, Lanzhou 730070, China; 3 Key Laboratory of New Animal Drug Project of Gansu Province, Lanzhou 730050, China; 4 Key Laboratory of Veterinary Pharmaceutical Development, Ministry of Agriculture and Rural Affairs, Lanzhou 730050, China

**Keywords:** *Acinetobacter*, plasmids, antibiotic resistance genes, *tet*(X3), *tet*(X4)

## Abstract

The coexistence of antibiotic resistance genes (ARGs), particularly those conferring resistance to last-resort antibiotics, is increasingly present in lesser-studied bacterial species. Tigecycline is currently one of the last important barriers in the treatment of carbapenem-resistant bacterial infections, whose resistance gene, *tet*(X), is prevalent across multiple bacterial genera, but the coexistence of *tet*(X3) and *tet*(X4) in *Acinetobacter* sp. is rarely observed. Here, we report a strain co-harborin*g* the chromosomal *tet*(X3) and plasmid-borne *tet*(X4) isolated from a commercial beef cattle farm in Henan province, China. The strain exhibited resistance to ampicillin, gentamicin, chloramphenicol, sulfamethoxazole, tetracycline, doxycycline, tigecycline, and omadacycline. Based on whole-genome sequencing (WGS), the strain was identified as *A. indicus* using Average Nucleotide Identity (ANI) and digital DNA–DNA hybridization (dDDH). Chromosomal *tet*(X3) was identified in the genetic context, IS*Vsa3*-*XerD*-*tet*(X3)-*res*-IS*Vsa3*. The plasmid-located *tet*(X4) with the genetic context, IS*Vsa3*-*abh*-*tet*(X4)-IS*Vsa3*, and 14 additional resistance genes were located in multiple p*dif* modules. Two different typing methods, the Rep-based strategy (designed for *A. baumanii*) and MOB-typer, identified the *tet*(X4)-positive plasmid as GR31 and rep_cluster_1656, respectively. Conjugation assay failed to observe the transfer of the *tet*(X4)-positive plasmid into recipients, *E. coli* J53 and *Salmonella* LGJ2. The co-occurrence of *tet*(X3) and *tet*(X4) in *Acinetobacter* may suggest a risk of rapid dissemination of tigecycline resistance and the hidden presence of numerous undetected bacteria co-carrying high-risk ARGs in the agroecological system, both of which should cause particular concern.

## 1. Introduction

Tigecycline, a broad-spectrum modified minocycline derivative, is considered a drug of last resort against multidrug-resistant bacteria, especially carbapenem-resistant bacteria (CRB) [1]. However, the emergence of plasmid-mediated tigecycline resistance gene *tet*(X) undermined this key line of defense. In particular, the novel tigecycline resistance genes *tet*(X3) and *tet*(X4) have recently been identified in *Acinetobacter* and *Enterobacteriaceae* from animals and humans [2]. They exhibit a more potent tigecycline resistance phenotype than the earlier-detected variants (such as *tet*(X) and *tet*(X2)) and have rapidly become the most epidemic variant within the *tet*(X) family [3]. These genes were first discovered in China in 2018 [1] and have been detected as widely distributed across dozens of provinces, including Liaoning, Hebei, Henan, Shanxi, Jiangsu, Zhejiang, Shaanxi, Gansu, Sichuan, Hunan, Hubei, Guangxi, and Guangdong [4,5]. In addition to China, this gene has been widely distributed across dozens of countries worldwide, including Canada, Thailand, the Republic of Korea, Iran, South Africa, Turkey, Iraq, the United Kingdom, Pakistan, Norway, and Singapore [6,7]. So far, human-, animal-, and environment-derived *tet*(X3) and *tet*(X4) have been reported in hospitals [8], farms [4,5], slaughterhouses [9], and supermarkets [10].

*Acinetobacter* comprises a complex, heterogeneous group of bacteria and can colonize the skin, throat, respiratory, and digestive tracts of humans and animals [11]. *Acinetobacter* can spread through food production, processing, and storage; through the hands of healthcare personnel; and through cross-contamination of medical devices, making it a significant contributor to nosocomial infections [12]. In recent years, *Acinetobacter* has attracted increasing public attention due to its frequent association with genes conferring resistance to last-resort antibiotics [13,14,15]. Furthermore, a new transfer mechanism of antibiotic resistance, associated with intracellular mobilization, has been identified in *Acinetobacter* [16,17]. This mechanism involves the p*dif*-ARG module flanked by XerCD site-specific recombination sites, which is mediated by the XerCD site-specific recombination (XerCD SSR) system [18]. XerC and XerD are encoded by numerous bacteria, usually in pairs. They are homologous recombinases (tyrosine recombinase family), which catalyze the cleavage of two consecutive pairs of DNA strands and exchange with a restriction site, *dif*, located in the terminus region of the chromosome [18]. Typically, the *dif* site is a 28 bp site consisting of two inverted repeat 11 bp Xer-binding motifs (the left and right regions of C/D and D/C), separated by a six bp interval called the central region. A monomer of XerC and XerD each binds to an 11 bp semi-binding site [19]. Usually, a single *dif* site is involved in dimeric chromosome resolution [19]. The *dif* sites in plasmids are called p*dif* sites, appear multiple times in a plasmid, and are considered to be associated with the transfer of resistance genes [20].

Here, we exhibit the multidrug-resistant *A. indicus* co-harboring chromosomal *tet*(X3) and plasmid-borne *tet*(X4) isolated from a commercial beef cattle farm in the Henan province of China.

## 2. Methods and Materials

**Sampling and microbial identification.** Cattle feces samples were collected from a commercial beef cattle farm in Henan province, China, in 2023. Specimens were preserved and transported in Brain Heart Infusion (BHI) broth (HuanKai Microbial, Guangzhou, China) to maintain microbial viability. Strain was isolated from MacConkey (HuanKai Microbial, Guangzhou, China) plates supplemented with tigecycline (2 mg/L, Solarbio, Beijing, China) with incubation for 16 h at 37 °C. A universal primer pair [1] was used to amplify the *tet*(X) gene in presumptive positive strains, followed by Sanger sequencing confirmation.

**Whole-genome sequencing.** Long-read sequencing was performed on the Oxford Nanopore platform. Briefly, the genome was sequenced using the Oxford Nanopore and DNBSEQ (short-read sequencing) platforms (Beijing Genomics Institution, Beijing, China). The corrected reads were generated by hybrid assembly in combination with DNBSEQ short reads. The assembled genome was checked for completeness and contamination using CheckM (v 1.2.3). The draft genome was assembled using SPAdes (v 3.9.0) with default parameters. Genome assembly and plasmid circularization detection were performed using Canu (v 2.2) with default parameters.

**Bacterial species identification.** Bacterial species identification was carried out on the JSpeciesWS and Deutsche Sammlung von Mikroorganismen und Zellkulturen (DSMZ) platforms using tetra correlation search (TCS) and type strain genome server (TYGS). The Average Nucleotide Identity (ANI) and digital DNA–DNA hybridization (dDDH) were calculated on the EZBioCloud and the DSMZ platforms using the OrthoANIu algorithm and genome-to-genome distance calculator (GGDC).

**Antimicrobial susceptibility testing.** The minimum inhibitory concentration (MIC) of 18 antibiotics (meropenem, aztreonam, ampicillin, ceftazidime, cefepime, gentamicin, chloramphenicol, colistin, kanamycin, fosfomycin, ciprofloxacin, sulfamethoxazole, azithromycin, tetracycline, doxycycline, tigecycline, rifampin, and omadacycline) was determined using microdilution of Mueller-Hinton broth (Huan Kai Microbial, Guangzhou, China). Microdilution was performed according to the method recommended by Clinical and Laboratory Standards Institute (CLSI). The testing concentration range is 2 mg/L to 1024 mg/L. Results were interpreted according to CLSI documents M100-S34 (2024) [5]. Since there is no established breakpoint for the tigecycline and omadacycline resistance of *Acinetobacter*, their breakpoints were determined by FDA-defined interpretive criteria for *Enterobacteriaceae* (R ≥ 8 mg/L for tigecycline and R ≥ 16 mg/L for omadacycline). *E. coli* ATCC25922 was used as a quality control for antimicrobial susceptibility testing, and the quality control range for ATCC25922 was based on the CLSI criterion. An independent colony was picked for testing, and MIC determinations were performed in triplicate.

**Conjugation assay.** Conjugation assays were performed using the filter mating method. *E. coli* J53 (sodium azide-resistant) and *Salmonella* LGJ2 (rifampicin-resistant) were used as recipients to observe the transfer of *tet*(X) to *Enterobacteriaceae*. Bacteria solution with a donor-to-recipient ratio of 10:1 (*tet*(X)-positive strain to J53 or LGJ2) was incubated on a 0.22 μm filter membrane at 35 °C for 16 h, then moved to MacConkey plates supplemented with antibiotics for which the donor (4 mg/L tigecycline) and recipient (100 mg/L sodium azide or 200 mg/L rifampicin) are resistant. The donor-to-recipient ratios of 5:1 and 15:1 were supplemented to test. At least three attempts were made for each parameter, and three parallel experiments were made for each attempt.

**Phylogenetic tree construction.** A maximum-likelihood (ML) phylogenetic tree based on core-genome single-nucleotide polymorphisms (cgSNP) was constructed using the *A. indicus* genome T63 and all *A. indicus* genomes stored in the NCBI Genome Database. SNPs were yielded using Snippy (v 4.6.0) with default parameters, and strain T63 was used as the reference genome. Each genome produced a core genome of about 60 kb. Snippy-core (v 4.6.0) was used to combine multiple Snippy outputs into a core SNP alignment. SNP distance matrix (v 0.8.2) was used to compute distance in SNPs between all sequences. Recombination filtering was not applied because it prevented a sufficient number of core genomes from being yielded. The ML phylogenetic tree was built with FastTree (v 2.2) and further visualized in iTOL (v 7) to highlight the features of each strain.

**Bioinformatics analysis.** ResFinder (v 4.6.0) was used to screen all known acquired antibiotic resistance genes (ARGs). The ARG identification threshold was set to 90%, and the minimum length was set to 80%. MOB-typer was used to search replicon, *oriT*, relaxase, T4CP, and T4SS, as well as to type plasmid. Furthermore, another plasmid typing based on a Rep-based strategy (designed for *A. baumannii*) [21,22] was performed using SnapGene (GR BLASTn). P*dif* sites were searched on the p*dif*Finder platform. Genome annotation was performed using the RAST genome annotation service, with further manual correction using ORFfinder, UniProt, and ISFinder. The linear genomic comparison was created using Easyfig (v 2.2.5).

## 3. Results

In August 2023, we isolated a tigecycline-resistant strain, T63, from a cattle fecal sample collected at a commercial beef cattle farm in Henan province, China. PCR amplification and Sanger sequencing verified that the strain carried the *tet*(X) gene.

**The identification of bacterial species.** The strain was sequenced using WGS on the Oxford Nanopore platform (long-read sequencing technology). The assembled genome was confirmed to be 99.89% completeness and 0.68% contamination. Current standards require ANI > 95% and dDDH > 70% to robustly assign a novel strain to a species. Thus, the genome was searched on the JSpeciesWS and DSMZ platforms using TCS and TYGS. Results revealed that strain T63 best matched GCF_000369465.1, GCF_000488255.1, GCF_000830155.1, and GCF_000413875.1. These genomes registered as *Acinetobacter indicus*. Subsequently, the ANI and dDDH values between genome T63 and these genomes were analyzed on the EZBioCloud and DSMZ platforms using the OrthoANIu algorithm and DDGC. Results found that genome T63 exhibited > 95% ANI and >70% dDDH with each of these genomes, with the highest ANI and dDDH values with GCF_000369465.1 (ANI: 97.98%, dDDH: 82.1%). Based on the above analyses, strain T63 was identified as *A*. *indicus* within the *Acinetobacter* genus under family *Moraxellaceae* (NCBI taxonomy database).

**Genetic diversity analysis.** The 408X coverage was obtained for short-read sequencing, and the 344X coverage was obtained for long-read sequencing. The WGS of strain T63 yielded one circular chromosome (2,986,742 bp, 45.9% GC content) and three circular plasmids (pT63-1: 116,245 bp, 41.8%; pT63-2: 13,463, 38.7%; pT63-3: 11,175, 35.1%) (Table 1). The GC contents of pT63-2 and pT63-3 were significantly lower than that of the chromosome, hinting that they originated from different hosts. GC content can serve as a marker to assess the origin of plasmids, indicating potential horizontal transfer [23]. Tigecycline resistance genes, *tet*(X3) and *tet*(X4), were located in the chromosome and plasmid (pT63-1) of strain T63, respectively (Table 1). A total of 19 resistance genes were identified in strain T63, among which 4/19 (23.53%) genes, including *tet*(X3), *aph(6)-Id*, *aph(3″)-Ib* and *sul2*, were located in the chromosome, and 2/19 (11.76%) genes, including *msr*(E) and *mph*(E) were located in the pT63-2 (Table 1). Notably, 17/19 (89.47%) genes, including *aac(3)-IId*, *aph(3′)-Ib*, *aph(3″)-Ib*, *aadA8*, *bla*_CARB-2_, *bla*_OXA-58_, *msr*(E), *mph*(E), *lnu*(G), *floR*, *sul2*, *sul3*, *tet*(X4), *tet*(39), *dfrA16*, *tmrB*, and *qacL* were located in the pT63-1 (Table 1). No resistance gene was identified in the pT63-3 (Table 1). The *tet*(X4)-positive plasmid (pT63-1) accounted for 89.47% of the resistant genes carried by the strain, reflecting co-selection-driven plasmid persistence.

**Antibiotic resistance phenotype.** The antimicrobial susceptibility testing showed that strain T63 was resistant to ampicillin (256 mg/L), gentamicin (8 mg/L), chloramphenicol (64 mg/L), sulfamethoxazole (1024 mg/L), tetracycline (64 mg/L), doxycycline (16 mg/L), tigecycline (8 mg/L), and omadacycline (32 mg/L) (Table 2). However, it was susceptible to meropenem, aztreonam, ceftazidime, cefepime, colistin, kanamycin, fosfomycin, ciprofloxacin, azithromycin, and rifampin (Table 2).

**Figure 1:**
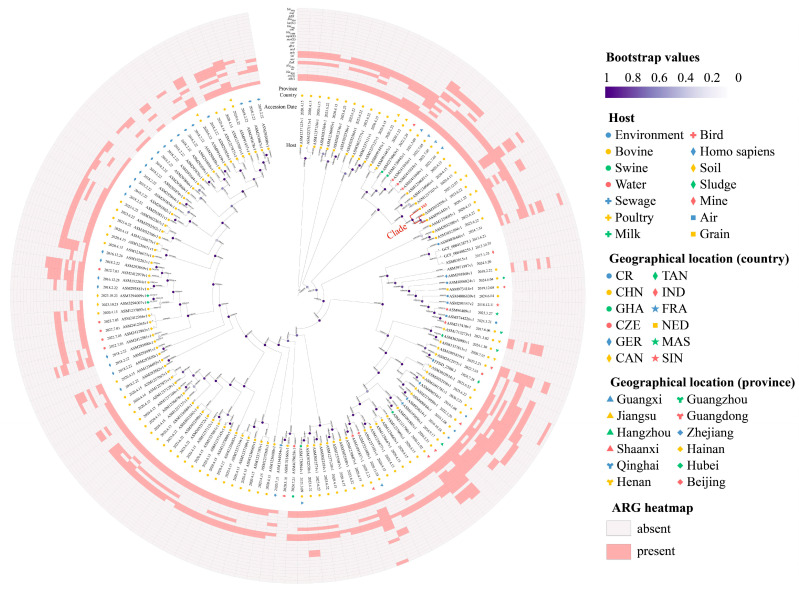
**An ML cgSNP-based phylogenetic tree based on all *A. indicus* genomes (including T63) stored in the NCBI database.** The clade including genome T63 is highlighted in red. Bootstrap values are shown as circles. Branch lengths are displayed as numbers on each branch of this tree.

**Phylogenetic analysis.** There were only 138 *A. indicus* genomes stored in the NCBI genome database. These genomes and T63 were used to build an ML circular phylogenetic tree based on cgSNPs to understand the genetic similarity of strain T63. The analysis found that strain T63 exhibited genetic similarity with ASM3032558v1 and ASM991443v1, which was characterized by the low level of core genome diversity that was reflected by a limited number of single-nucleotide polymorphisms (SNPs ≤ 340) (Figure 1). These strains showed similar isolation sources (bovine) and geographical locations (China) to strain T63 (Figure 1). Notably, these strains and strain T63 exhibited a similar resistance gene profile. They carried *tet*(X3), *sul2*, *aph(3″)-Ib* and *aph(6)-Id*, particularly ASM991443v1 and T63 additionally carrying *aac(3)-IId*, *bla*_OXA58_, *floR*, *msr*(E) and *mph*(E).

**Plasmidome analysis**. The plasmid typing based on MOB-Typer revealed that pT63-1-*tet*(X4) was typed to rep_cluster_1656, pT63-2 to rep_cluster_1254, and pT63-3 to an unidentified type (Table 1). The *ori*T of these plasmids could not be identified. The relaxases of pT63-1-*tet*(X4) and pT63-2 were identified as MOBP and MOBQ, which belonged to the MOB family (Table 1). The pT63-1-*tet*(X4) and pT63-2 were predicted as mobilizable, and pT63-3 was predicted as non-mobilizable (Table 1). Subsequently, plasmid typing based on a Rep-based strategy (designed for *A. baumannii*) revealed that pT63-1-*tet*(X4) best matched GR31 (99.66% nucleotide identity and 100% coverage), a RepB-family plasmid replication initiator protein. The remaining plasmids did not best match the identified GRs of the Rep-based strategy (<50% nucleotide identity and coverage). Conjugation assay demonstrated that the *tet*(X4)-positive plasmid could not be transferred to recipients, *E. coli* J53 or *Salmonella* LGJ2, despite multiple experimental parameters and repeated attempts employed.

**Figure 2:**
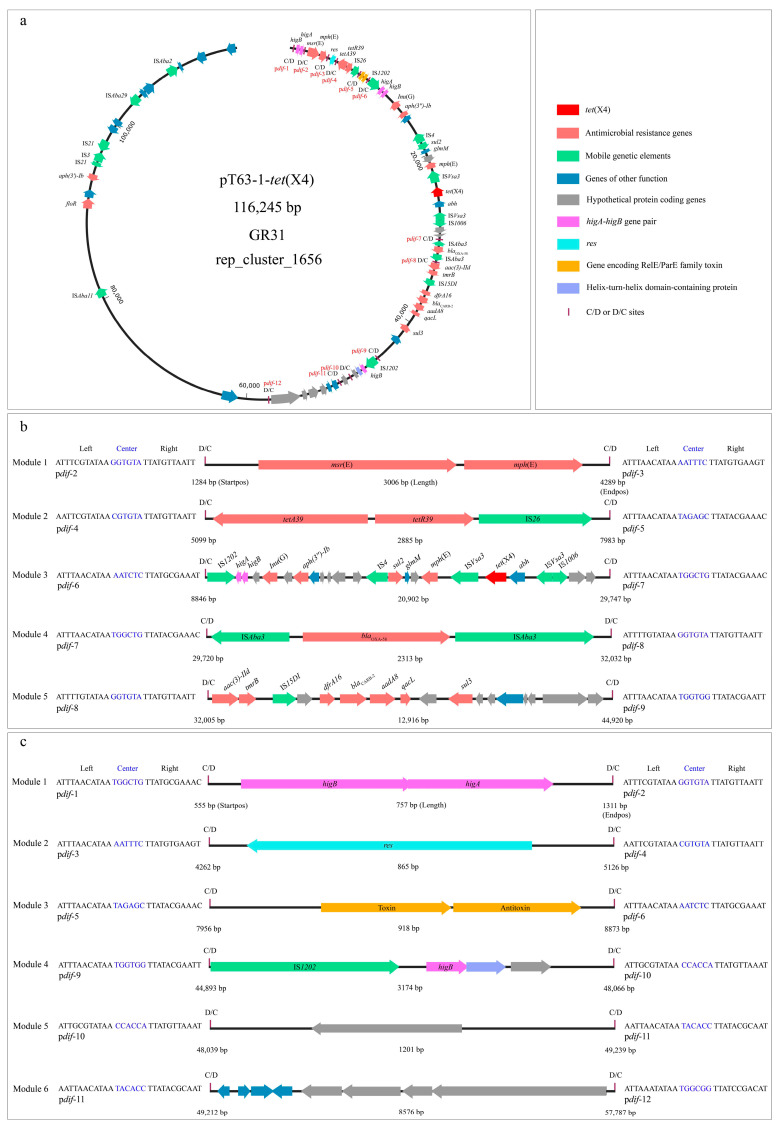
**Genetic characteristics of the *tet*(X4)-positive plasmid based on the ONT long-read sequencing.** (**a**): Circular structure of *tet*(X4)-positive plasmid includes the location of ARGs, mobile genetic elements, genes of other functions, as well as p*dif* sites and the modules they build. (**b**): Genetic contexts of five p*dif*-ARG modules. (**c**): Genetic contexts of the p*dif* modules containing non-resistant genes. The p*dif* sites building p*dif* modules are listed on both sides of these modules. The C/D and D/C are a 28 bp site consisting of two inverted repeat 11 bp XerC and XerD binding motifs (the left and the right regions) separated by a six bp interval (central region).

**Figure 3:**
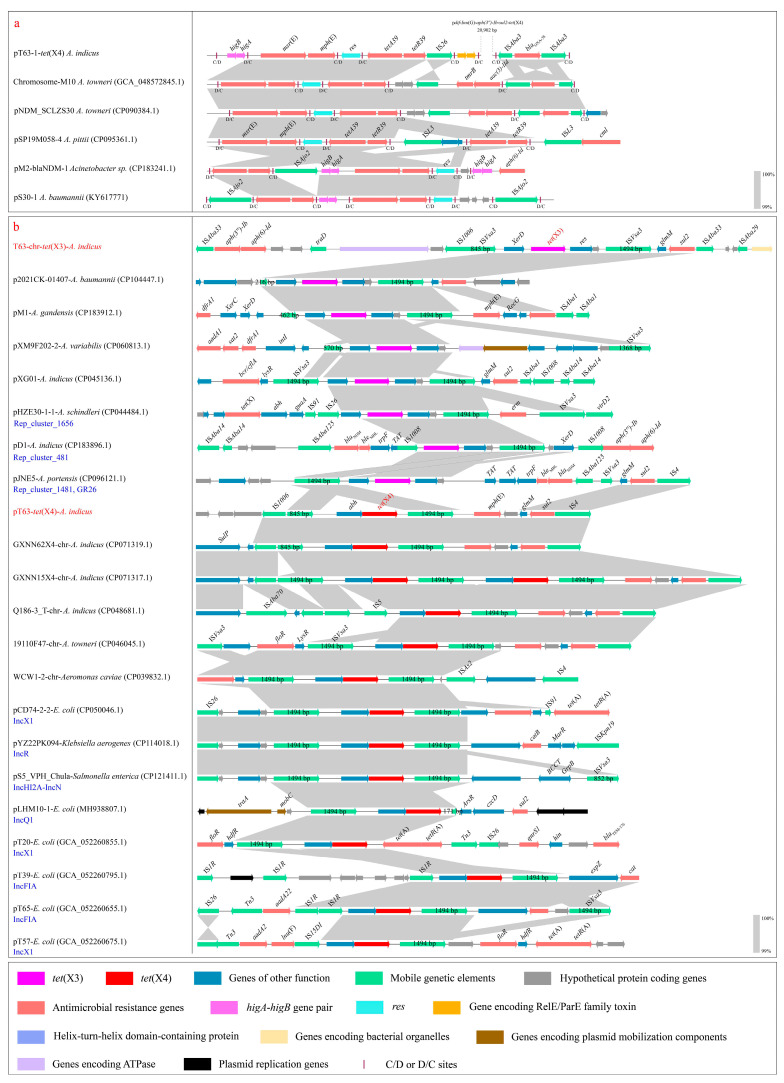
**Linear comparison based on the p*dif*-ARG modules and the genetic contexts of *tet*(X3) and *tet*(X4) identified in strain T63.** (**a**): The comparison analysis of p*dif*-*msr*(E)-*mph*(E), p*dif*-*bla*_OXA-58_, and p*dif*-*tetA39*-*tetR39* modules. (**b**): Linear comparison of the genetic contexts of *tet*(X3) and *tet*(X4), as well as their upstream and downstream regions.

**P*dif*-ARG module analysis.** The analysis based on p*dif*Finder revealed a p*dif* module island composed of 12 p*dif* sites (Figure 2a–c), containing 11 consecutively arranged p*dif* modules (Figure 2a). This special configuration inevitably leads to sharing the internal C/D or D/C sites to form two types of p*dif* modules, one flanked by a C/D and a D/C site, and the other type flanked by a D/C and a C/D site (Figure 2a). These modules included five p*dif*-ARG modules, p*dif*-*msr*(E)-*mph*(E) (length: 3006 bp), p*dif*-*tetA39*-*tetR39*-IS*26* (2885 bp), p*dif*-*Inu*(G)-*aph(3″)-Ib-sul2-tet*(X4) (20,902 bp), p*dif*-*bla*_OXA-58_ (2313 bp) and p*dif*-*aac(3)-IId*-*dfrA16*-*bla*_CARB-2_-*aadA8*-*sul3* (12,916 bp) (Figure 2b). Among these modules, p*dif*-*Inu*(G)-*aph(3″)-Ib-sul2-tet*(X4) and the p*dif*-*aac(3)-IId*-*dfrA16*-*bla*_CARB-2_-*aadA8*-*sul3* modules have not been reported, with no similar segments (<70% identity and coverage) identified in the NCBI database. P*dif*-*msr*(E)-*mph*(E), p*dif*-*bla*_OXA-58_, and p*dif*-*tetA39*-*tetR39* have been described in previous studies, and similar segments (>99% identity and coverage) could be identified in the NCBI database. Thus, based on the high similarity (>99% identity and coverage), multiple segments containing these modules from chromosomes and plasmids of *Acinetobacter* were used to compare with those in the *tet*(X4)-positive plasmid (Figure 3a). The analysis found that p*dif*-*msr*(E)-*mph*(E) and p*dif*-*bla*_OXA-58_ modules were highly conserved (>99% identity and coverage) in the chromosomes and plasmids of various *Acinetobacter* (*A. pittii*, *A. baumannii*, and *A. towneri*) (Figure 3a). However, the p*dif*-*tetA39*-*tetR39*-IS*26* module in pT63-1-*tet*(X4) observably differed from the typical p*dif*-*tetA39*-*tetR39* module [20] due to the IS*26* downstream of *tetR39* (Figure 3a). A similar characteristic was identified in the pM2-*bla*_NDM-1_ (CP183241.1) [15], in which the p*dif*-IS*Ajo2*-*higA*-*higB*-*msr*(E)-*mph*(E) module was significantly different from the typical module only formed by *msr*(E)-*mph*(E) (Figure 3a).

In addition to the p*dif*-ARG module, six p*dif* modules carrying genes encoding other functional proteins or hypothetical proteins were identified. Module one (757 bp) harbored a gene pair of *higA*-*higB*, which belongs to Type II toxin–antitoxin systems. Module two (865 bp) carried the *res* gene encoding resolvase (Figure 2c). Module three (918 bp) contained genes encoding the RelE/ParE toxin and RelB/ParD antitoxin (Figure 2c). Notably, the above modules typically appear as independent units and are closely adjacent to the Xer-ARG module (Figure 3a). Module four (3174 bp) carried IS*1202*, *higB*, and the genes encoding helix-turn-helix domain-containing protein and hypothetical protein (Figure 2c). The orphaned toxin (*higB*) may interact with the antitoxin located at another site on the plasmid. Module five (1201 bp) only contained a gene encoding a hypothetical protein (Figure 2c). Module six (8576 bp) contained genes encoding recombinase, DUF1778 domain-containing protein, GNAT family N-acetyltransferase, and hypothetical proteins (Figure 2c).

**The genetic contexts of chromosomal *tet*(X3).** *Tet*(X3) was located within a typical genetic context (5949 bp), IS*Vsa3*-*XerD*-*tet*(X3)-*res*-IS*Vsa3*, in the chromosome of strain T63 (Figure 3b). BLASTn analysis of the center region, *XerD*-*tet*(X3)-*res*, in the NCBI database found that this region was generally located on chromosomes and plasmids of various *Acinetobacter* species. Based on the high similarity (>99.00% identity and coverage) of this region, multiple segments (long-read sequencing) containing the upstream and downstream regions of the *tet*(X3) genetic context from *Acinetobacter* plasmids (*A. baumannii*, *A. gandensis*, *A. variabilis*, *A. schindleri*, and *A. portensis*) were used to compare with those in the *tet*(X3)-positive chromosome (Figure 3b). Among these plasmids, 4/7 plasmids could not be typed using the MOB-Typer and Rep-based strategy (Figure 3b). The remaining plasmids (3/7) were typed as Rep_cluster_1656, Rep_cluster_1481, and Rep_cluster_481 using MOB-Typer (Figure 3b). Only one plasmid was typed as GR26 (>99.00% identity and coverage) using the Rep-based strategy (Figure 3b). The typical *tet*(X3) genetic context was two copies of IS*Vsa3* flanking *XerD*-*tet*(X3)-*res*, which were invariably in the same orientation (Figure 3b). Usually, in this context, upstream IS*Vsa3* was either intact (1494 bp) or truncated (216–845 bp), but downstream IS*Vsa3* was commonly intact (1494 bp) (Figure 3b). Furthermore, various other genetic contexts could also be observed, such as a single IS*Vsa3* (intact and truncated) located upstream or downstream of *tet*(X3) (pXM9F202-2 and pJNE5); the upstream or downstream IS*Vsa3* replaced by other IS elements like IS*26* or IS*1008* (pHZE30-1-1 and pD1) (Figure 3b). Notably, a structure change in the center region, *XerD*-*tet*(X3)-*res*, was observed in pD1, in which *XerD* shifted to downstream of intact IS*Vsa3*, and only *tet*(X3)-*res* was conserved (Figure 3b). The upstream and downstream regions of the *tet*(X3) genetic context exhibited significant differences, but multiple IS elements and resistance genes were commonly adjacent to the *tet*(X3) genetic context (Figure 3b).

**The genetic contexts of plasmid-mediated *tet*(X4).** *Tet*(X4) was located in a genetic context (5440 bp) with the gene arrangement, IS*Vsa3*-*abh*-*tet*(X4)-IS*Vsa3*, in the plasmid of *A*. *indicus* T63 (Figure 2a). An IS*1006* in the opposite direction to IS*Vsa3* was closely adjacent to the upstream IS*Vsa3* (Figure 2a). *Mph*(E)-*glmM*-*sul2*-IS*4* was located in the region adjacent to downstream IS*Vsa3* (Figure 2a). Analysis of the center region, *abh*-*tet*(X4), in the NCBI database found that it was typically carried by chromosomes and plasmids of *Enterobacteriaceae*, as well as the chromosomes of *Acinetobacter*. However, it was rarely located in the *Acinetobacter* plasmids. Thus, based on the high similarity (>99.00% identity and coverage) of this region, multiple segments (long-read sequencing) containing the upstream and downstream regions of *tet*(X4) genetic context from these typical bacterial vehicles (chromosomes: *A. indicus*, *A. towneri*, and *Aeromonas caviae*; plasmids: *E. coli* (IncX1, IncQ1, IncFIA), *Klebsiella aerogenes* (IncR), and *Salmonella enterica* (IncHI2A-IncN) were used to compare with those in the *tet*(X4)-positive *Acinetobacter* plasmid (Figure 3b). The analysis revealed that the center region, *abh*-*tet*(X4), was highly conserved in *Acinetobacter* and *Enterobacteriaceae* (Figure 3b). Similarly to the *tet*(X3) genetic context, the diversity of *tet*(X4) genetic context was mainly mediated by the upstream and downstream IS*Vsa3* of *tet*(X4), with the following configurations: two intact copies of IS*Vsa3* flanked *tet*(X4); an intact and an truncated IS*Vsa3* flanked *tet*(X4); a replacement of the upstream or downstream IS*Vsa3* by other IS elements, such as IS*1R* or IS*15DI*; the absence of the upstream or downstream IS*Vsa3* (Figure 3b). The genes located within the upstream and downstream regions of the *tet*(X4) genetic context in *Enterobacteriaceae* plasmids were complex and versatile. In contrast, the genes located within the upstream and downstream regions of the *tet*(X4) genetic context in *Acinetobacter* were relatively stable and conserved, such as the high occurrence frequency of *sulP* and IS*1006* in the upstream region; the high conservation of *mph*(E)-*glmM*-*sul2*-IS*4* in the downstream region (Figure 3b). This hints that the transmission of *tet*(X4) in *Enterobacteriaceae* may be more frequent than that in *Acinetobacter*. Furthermore, the gene pair, *glmM*-*sul2*, emerged frequently in the downstream regions of genetic contexts for *tet*(X3) and *tet*(X4) and was conserved across *Acinetobacter* chromosomes and plasmids (Figure 3b).

## 4. Discussion

Livestock is recognized as a critical reservoir for tigecycline-resistant bacteria [4,5], yet investigations into bovine production remain limited. The current study reveals that bovine-derived *A*. *indicus* co-harbors the chromosomal *tet*(X3) and the plasmid-located *tet*(X4). Tigecycline is regarded as a crucial last-resort antibiotic in human medicine, but its use in food-producing animals is not authorized worldwide [4]. It is noteworthy that the detection rate of the *tet*(X) correlates positively with the frequency of tetracycline antibiotic use, indicating that tetracycline utilization is a driving force promoting the enrichment and dissemination of the *tet*(X) [4,24]. In the livestock industry, older tetracycline antibiotics, such as chlortetracycline and oxytetracycline, remain extensively employed as feed additives or therapeutic drugs, which may drive the co-occurrence of *tet*(X3) and *tet*(X4). The *tet*(X3) is initially detected in the *A. baumanii* plasmid (p34AB), and *tet*(X4) is first identified in the *E. coli* plasmid (p47EC) [1]. Although the rapid transmission of these genes, *tet*(X4), exhibits a more formidable dissemination capability [25]. Compared with the typical location of *tet*(X3) in the chromosomes and plasmids of *Acinetobacter* [26,27], *tet*(X4) is rarely carried by the *Acinetobacter* plasmid. The *tet*(X4) can be identified in the *Acinetobacter* chromosome, but it is commonly located in the chromosomes and plasmids of *Enterobacteriaceae*, such as *Escherichia* [4,5], *Klebsiella* [28], and *Enterobacter* [29]. *Enterobacteriaceae* constitute typical flora within animal gut systems and environmental habitats, thereby subjecting them to more direct and persistent exposure to the selective pressure exerted by antibiotics. Although *Acinetobacter*, as a commensal bacterium, can colonize the digestive system together with *Enterobacteriaceae*, its colonization level is significantly lower than that of *Enterobacteriaceae* [11]. Moreover, current research on antibiotic resistance has focused on *Enterobacteriaceae*. However, *Acinetobacter*, a heterogeneous bacterial genus widely distributed in human and animal communities with robust environmental adaptability and survival capabilities [30], has been neglected (reflected in a limited number of genomes in the NCBI database), which may lead to an underestimation of the spread of *tet*(X3) and *tet*(X4) in this genus.

*Tet*(X4) is typically located in a wide variety of Inc plasmids, including single replicon and multireplicon plasmids, such as IncX1, IncFIA, IncHI1A, IncHI1B, IncR, IncN, IncHI1A-IncR, IncX1-IncN, IncX1-IncR, etc. [5]. Inc plasmids are considered the dominant vector for the spread of *tet*(X4) and contribute to the global epidemic of *tet*(X4) [24,31]. These plasmids are usually seen in *Enterobacteriaceae* and emphasize the plasmid replication and the coexistence of multiple replicons, with a diversified host range. They can carry resistance genes and facilitate the horizontal transfer of resistance genes among different bacterial genera via conjugation [24]. Inc plasmids commonly exhibit optimal replication and maintenance fitness within *Enterobacteriaceae*. In contrast to Inc plasmids, *Acinetobacter* plasmids exhibit genus-specificity and primarily adapt, persist, and evolve within the genus *Acinetobacter*, with a narrow host range [32]. This study failed to observe the transfer of the *tet*(X4)-positive *Acinetobacter* plasmid via conjugation assays. Meanwhile, the MOB-Typer analysis indicated that this plasmid lacked *ori*T and was predicted to be mobilizable rather than conjugative. Compared to the commonly observed transferability of Inc plasmids, *Acinetobacter* plasmids were rarely reported to be conjugative [33]. However, this is inconsistent with established biological principles: (1) conjugative plasmids serve as fundamental drivers of bacterial evolution by facilitating the dissemination of antibiotic resistance genes, heavy metal tolerance determinants, and virulence factors, which enables bacterial adaptation to diverse environmental and selective pressures [34]; (2) a wide variety of plasmids, whether conjugative or not, have been observed in *Acinetobacter* genomes [35]. Laboratory systems may lack key microbial ecological drivers, such as nutrient gradients, multispecies competitive dynamics, and stress-induced epigenetic regulatory pathways, resulting in the rarely observed transferability of *Acinetobacter* plasmids. Furthermore, recent studies revealed that natural transformation, a horizontal gene transfer mechanism highlighted in *A. baumannii*, enables highly efficient interbacterial transfer of genetic elements conferring resistance to last-line carbapenem antibiotics. This should be considered for the other gene conferring resistance to last-resort antibiotics and the other species of *Acinetobacter* [36].

The dissemination of *tet*(X3) and *tet*(X4) critically depends on the flanking insertion sequence, IS*Vsa3*. This element can mobilize adjacent genes via a rolling-circle transposition mechanism [1]. Most IS elements typically require two copies (one of which must be intact) to complete transposition, such as the transposition of *mcr-1* mediated by IS*Apl1* [37], whereas IS*Vsa3* can achieve transposition of adjacent DNA sequences using a single copy [38]. In addition to the *tet*(X) family, IS*Vsa3* is associated with multiple ARGs, such as *floR*, *tet(A)*, *aph(6)-Id*, *aph(3″)-Ib*, and *sul2* [39]. Thus, monitoring IS*Vsa3* is critical for understanding the distribution and spread of ARGs, particularly *tet*(X). The various *tet*(X3) and *tet*(X4) genetic contexts have been found in *Enterobacteriaceae* and *Acinetobacter*, which may be attributed to the dynamic IS elements adjacent to IS*Vsa3* and from recurrent rearrangement events. The upstream and downstream regions of the *tet*(X3) genetic context in *Acinetobacter* exhibit more complexity and diversity than that of the *tet*(X4) genetic context, suggesting that the transmission of *tet*(X3) in this genus may be more dynamic than that of *tet*(X4). Furthermore, the *glmM*-*sul2* gene pair is potentially associated with the transfer of *tet*(X3) and *tet*(X4) in *Acinetobacter*, as it is frequently located in the downstream regions of the genetic contexts of *tet*(X3) and *tet*(X4) within the chromosomes and plasmids of this genus.

A total of 15 resistance genes were identified in the *tet*(X4)-positive plasmid, of which 13/15 genes were located in the p*dif* modules. Previous studies [40] indicated that the p*dif* modules identified in *Acinetobacter* have a genus-specific characteristic. Among these modules, p*dif*-*msr*(E)-*mph*(E) and p*dif*-*bla*_OXA-58_ modules were highly conserved (>99% identity and coverage) with the Xer-ARG modules identified in the chromosomes and plasmids of *Acinetobacter* from the GenBank database, suggesting their potential mobilization. P*dif*-*tetA39*-*tetR39*-IS*26* module exhibited a difference with the typical module only formed by *tetA39*-*tetR39* due to the presence of IS*26*. A similar characteristic is also observed in p*dif*-IS*Ajo2*-*higA*-*higB*-*msr*(E)-*mph*(E) module [15], indicating that the elements located in the p*dif* module are variable and dynamic. P*dif*-*Inu*(G)-*aph(3*″*)-Ib-sul2-tet*(X4) module has not been matched in the NCBI database. However, an intact *tet*(X4) genetic context was identified in this module, which may contribute to the effective expression of tigecycline resistance and the maintenance of *tet*(X4) transfer capability [15]. The genes for which resistance phenotypes were tested exhibited resistance to the corresponding antibiotics. Among these, some genes are located in p*dif* modules, suggesting that these modules can perform their functions in antibiotic resistance.

Despite the findings, this study has several limitations. First, although antibiotic resistance phenotypes and genotypes were tested, it was not determined which gene was responsible when multiple genes were associated with the resistance phenotype. Second, the current inadequate plasmid typing methods constrain the study of *Acinetobacter* resistance. Inc testing is only suitable for *Enterobacteriaceae* plasmids, not for *Acinetobacter* plasmids [32]. In this study, we employed the MOB-typer and Rep-based strategy (designed for *A. baumannii* plasmids) [21,22], but these methods only classify plasmids into distinct Rep clusters and are not precise enough to cover the full range of plasmids. Furthermore, MOB-typer is insufficient to cover various elements, such as Rep, oriT, and relaxase, in *Acinetobacter* plasmids. Therefore, the predicted mobility should only be considered as a reference. Although a novel typing method for the *Acinetobacter* plasmid is needed, the extremely limited genomic data may delay its development. Third, phenotypic resistance research in *Acinetobacter* was constrained by the lack of interpretive breakpoints for most antibiotics in standard guidelines (CLSI, EUCAST, FDA). Fourth, FastTree is acceptable for rapid phylogenetic inference but is less accurate for fine-scale analyses. Fifth, the toxin–antitoxin systems observed are identical to those previously described in other sources, or are they only homologues, in which case the function or mode of action can only be inferred.

## 5. Conclusions

The current study exhibited that *A. indicus* co-carried the chromosomal *tet*(X3) and the plasmid-borne *tet*(X4). Genome analysis and antimicrobial susceptibility testing confirm the association between these genes and resistant phenotypes. The continuous emergence of lesser-studied bacterial species carrying genes conferring resistance to last-resort antibiotics, particularly those co-harboring these genes, is a global public health issue that cannot be ignored and, therefore, warrants more urgent attention.

## Figures and Tables

**Table 1:** Genomic characteristics include size (bp), GC content, ARGs, and plasmid typing using MOB typer and a Rep-based strategy. N/D: Not Detected.

		MOB Typer					Rep-Based Strategy	
T63	Size (bp) GC Content	Rep	Relaxase	*ori*T	Predicted Mobility	Best Match	Rep	ARGs (Identity %)
Chromosome	2,986,742 45.9%	-	-	-	-	-	-	*aph(6)-Id* (100%), *aph(3″)-Ib* (100%), *sul2* (100%), *tet*(X3) (99.91%)
pT63-1	116,245 41.8%	rep_cluster_1656	MOBP	N/D	Mobilizable	CP044019	GR31	*aac(3)-IId* (99.88%), *aph(3′)-Ib* (100%), *aph(3″)-Ib* (100%), *aadA8* (98.11%), *bla*_CARB-2_ (100%), *bla*_OXA-58_ (100%), *msr*(E) (100%), *mph*(E) (100%), *lnu*(G) (100%), *floR* (98.19%), *sul2* (100%), *sul3* (100%), *tet*(X4) (100%), *tet*(39) (99.82%), *dfrA16* (100%), *tmrB* (100%), *qacL* (100%)
pT63-2	13,463 38.7%	rep_cluster_1254	MOBQ	N/D	Mobilizable	LR026974	Unknown	*msr*(E) (100%), *mph*(E) (100%)
pT63-3	11,175 35.1%	N/D	N/D	N/D	Non mobilizable	CP033124	Unknown	N/D

**Table 2:** Antibiotic resistance genotype and phenotype (MICs against 18 antibiotics). N/D: Not Detected.

Antibiotics	MIC (mg/L)	Interpretation	ARGs
Meropenem (MEM)	<2	susceptible	N/D
Aztreonam (ATM)	4	susceptible	N/D
Ampicillin (AMP)	256	resistant	*bla*_CARB-2_, *bla*_OXA-58_
Ceftazidime (CAZ)	<2	susceptible	N/D
Cefepime (FEP)	<2	susceptible	N/D
Gentamicin (GEN)	8	resistant	*aac(3)-IId*
Chloramphenicol (CHL)	64	resistant	*floR*
Colistin (CL)	<2	susceptible	N/D
Kanamycin (KAN)	8	susceptible	N/D
Fosfomycin (FOS)	128	susceptible	N/D
Ciprofloxacin (CIP)	<2	susceptible	N/D
Sulfamethoxazole (SXT)	1024	resistant	*sul2*, *sul3*
Azithromycin (AZM)	8	susceptible	*msr*(E)
Tetracycline (TET)	64	resistant	*tet*(X3), *tet*(X4), *tet*(39)
Doxycycline (DOX)	16	resistant	*tet*(X3), *tet*(X4), *tet*(39)
Tigecycline (TGC)	8	resistant	*tet*(X3), *tet*(X4)
Rifampin (RIF)	<2	susceptible	N/D
Omadacycline (OMC)	32	resistant	*tet*(X3), *tet*(X4)
antibiotics not included in this experiment	-	-	*aph(6)-Id*, *aph(3′)-Ib*, *aph(3″)-Ib*, *aadA8b*, *mph*(E), *lnu*(G), *dfrA16*

## Data Availability

The assembled genome of strain T63 has been deposited in the NCBI database under BioProject accession no. PRJNA1343328.
